# Subtelomeric Chromatin in the Fission Yeast *S. pombe*

**DOI:** 10.3390/microorganisms9091977

**Published:** 2021-09-17

**Authors:** Rajesh K. Yadav, Atsushi Matsuda, Brandon R. Lowe, Yasushi Hiraoka, Janet F. Partridge

**Affiliations:** 1 Department of Biochemistry, All India Institute of Medical Sciences Patna, Patna PIN-801507, Bihar, India; rajesh.yadav@aiimspatna.org; 2 Advanced ICT Research Institute Kobe, National Institute of Information and Communications Technology, Kobe 651-2492, Japan; a.matsuda@nict.go.jp; 3 St. Jude Children’s Research Hospital, Memphis, TN 38105, USA; brandon.lowe@stjude.org; 4 Graduate School of Frontier Biosciences, Osaka University, Suita 565-0871, Japan

**Keywords:** heterochromatin, subtelomere, telomere, histone, acetyltransferase, methyltransferase, clustering, Shugoshin, shelterin, transcription

## Abstract

Telomeres play important roles in safeguarding the genome. The specialized repressive chromatin that assembles at telomeres and subtelomeric domains is key to this protective role. However, in many organisms, the repetitive nature of telomeric and subtelomeric sequences has hindered research efforts. The fission yeast *S. pombe* has provided an important model system for dissection of chromatin biology due to the relative ease of genetic manipulation and strong conservation of important regulatory proteins with higher eukaryotes. Telomeres and the telomere-binding shelterin complex are highly conserved with mammals, as is the assembly of constitutive heterochromatin at subtelomeres. In this review, we seek to summarize recent work detailing the assembly of distinct chromatin structures within subtelomeric domains in fission yeast. These include the heterochromatic SH subtelomeric domains, the telomere-associated sequences (TAS), and ST chromatin domains that assemble highly condensed chromatin clusters called knobs. Specifically, we review new insights into the sequence of subtelomeric domains, the distinct types of chromatin that assemble on these sequences and how histone H3 K36 modifications influence these chromatin structures. We address the interplay between the subdomains of chromatin structure and how subtelomeric chromatin is influenced by both the telomere-bound shelterin complexes and by euchromatic chromatin regulators internal to the subtelomeric domain. Finally, we demonstrate that telomere clustering, which is mediated via the condensed ST chromatin knob domains, does not depend on knob assembly within these domains but on Set2, which mediates H3K36 methylation.

## 1. Introduction

Telomeres, located at the ends of chromosomes, play critical roles in ensuring the maintenance of genomic stability. Not only do these structures provide a proteinaceous cap that protects the ends of the chromosomes from recombination but they also play key roles in ensuring proper chromosome movement during mitosis and meiosis. The function of telomeres and repetitive subtelomeric sequences is highly conserved amongst eukaryotes, although the exact DNA sequences are not highly conserved [1]. For many organisms, research into telomeric biology has been hampered by difficulty obtaining full genomic sequence information for these domains. Fission yeast *Schizosaccharomyces pombe* provides a highly useful genetically tractable system for exploring chromosome biology, and recently, the full sequence of subtelomeric domains has been defined. This review provides a non-exhaustive summary of the mechanisms underlying the assembly of distinct chromatin subtypes that assemble at subtelomeres in fission yeast, providing an overview of recent findings.

## 2. Nuclear Organization in *S. pombe*

*S. pombe* cells have a haploid genome consisting of three chromosomes: I, II, and III (5.6, 4.6, and 3.5 Mb, respectively) [2]. Centromeres of *S. pombe* are large satellite-rich domains that span 35–110 kb. The telomeres comprise ~300 bp of a repeat motif [G_2-6_TTAC[A]) [3,4]. Chr I and II have subtelomeric regions adjacent to the telomere repeat sequences at both ends; Chr III has rDNA repeats, which code for ribosomal RNA, replacing the subtelomeric regions in the *972h*- strain. Some strains possess partial subtelomeric sequences adjacent to the telomeres of Chr III [5,6]. The nuclear envelope (NE) remains intact throughout the cell cycle (closed mitosis) unlike higher animals and plants, in which the NE disassembles during mitosis (open mitosis). In the mitotic interphase, centromeres are associated with the spindle-pole body (SPB, a centrosome-equivalent structure in fungi), and telomeres are on the NE at the opposite side of SPB (reviewed in [7]). Telomeres are anchored to the NE through interaction between telomere protein Rap1, a component of shelterin, and NE protein Bqt4 [8,9]. Subtelomeres are attached to the NE through interaction between a chromatin remodeler Fft3 and LEM-domain nuclear membrane protein Man1 [10]. Fft3–Man1 interaction is also involved in NE anchoring of the long-terminal repeat (LTR) of retrotransposons [10].

**Figure 1:**
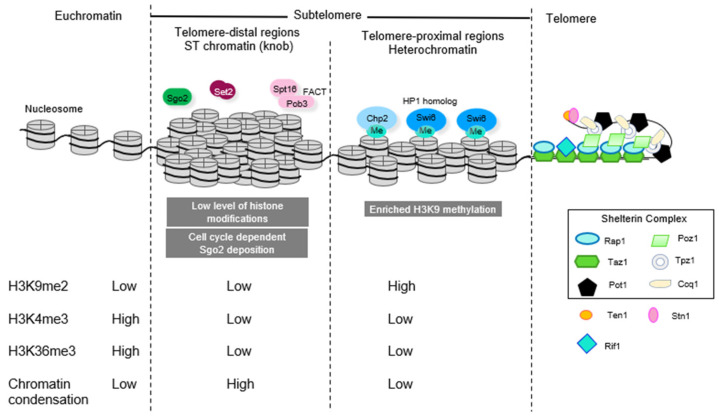
Subtelomeric chromatin domains consist of (1) heterochromatic telomere-proximal domains characterized by high levels of heterochromatic histone marks (H3K9me2) and a paucity of marks associated with transcriptional activity (H3K4me3) and bound by HP1 homolog proteins Swi6 and Chp2 and (2) telomere distal ST domains, where there is a paucity of histone modification and instead chromatin is highly condensed and associated with Shugoshin protein Sgo2 to form “knobs”.

Telomeric DNA, which spans about 300 nucleotides, is composed of double-stranded DNA repeats with a single-stranded overhang at the very end of the chromosome. Internal to this, the subtelomeric region spans about 100 kilobases to separate the telomeres from the euchromatic arms of the chromosomes (Figure 1). Telomeric and telomere-proximal regions form a specialized chromatin structure called the telosome [11]. Telosomes contains histones but are resistant to micrococcal nuclease digestion. This may stem from an unusual nucleosomal distribution caused by torsion from folding of the domain or possibly due to nucleosomal linker regions being protected from micrococcal nuclease digestion by telomeric proteins or the RNA that contributes to these structures [11]. While the telomere repeats constitute approximately 300 bp, the telosome constitutes 1 and 1.4 kb fragments and thus spans both telomeric and the adjacent subtelomeric regions.

## 3. Heterochromatin in *S. pombe*

**Figure 2:**
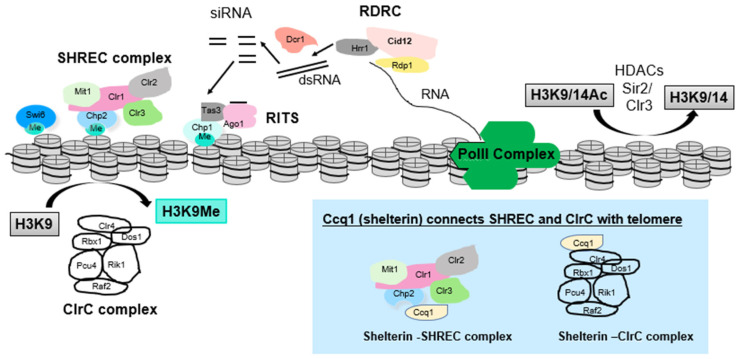
Complexes involved in heterochromatin assembly at subtelomeres in *S. pombe*. Complexes include (1) factors involved in the RNAi pathway (Dcr1, which generates siRNAs; RDRC complex, which amplifies RNAi signal; and RITS, which is the RNAi-effector complex and includes Ago1, which can bind siRNA to direct cleavage of nascent transcripts as well as bind to H3K9me), (2) ClrC complex, which directs H3K9 methylation, (3) HDACs such as Clr3, which resides in the SHREC complex that can remodel chromatin, and Sir2. At telomeres, (see blue box) the Ccq1 protein component of shelterin associates with SHREC and with ClrC to link heterochromatin assembly to telomere control. Curved arrows represent H3K9 methylation by ClrC complex and deacetylation of H3K9ac/K14ac by HDACs Sir2 and Clr3. Straight arrows depict RDRC-mediated generation of dsRNA from RNA Pol II transcript, cleavage of dsRNA by Dcr1 to form siRNAs and (not shown in detail) steps leading to eventual assembly of RITS loaded with siRNA.

Heterochromatin and euchromatin are made up of the same basic unit, the nucleosome, which consists of a histone octamer wrapped by 147 bp of DNA [12]. Histones are highly conserved basic proteins with N- and C- terminal tails that extend out from the nucleosome surface. Histones are subject to many types of post-translational modification, and heterochromatin and euchromatin are subject to different modifications of the histone tails that “mark” them to provide binding surfaces for additional chromatin proteins. Different types of post-translational modification can occur at many sites on the histone tails, leading to the idea of a complex regulatory “histone code” [13]. In general, histone acetylation correlates with transcriptional activity in euchromatic regions, whereas histone methylation can correlate with active or inactive states [14]. The *S. pombe* genome assembles constitutive heterochromatin at the pericentromeres, the telomeres, the mating-type locus, and rDNA loci that replace subtelomeric sequences on chromosome 3 [2]. The chromatin of these domains is methylated on histone H3K9 [15,16], which is a highly conserved mark of constitutive heterochromatin and serves as a recruitment site for chromodomain proteins of additional silencing assemblies such as Swi6 [HP1) and Chp1 and Chp2 proteins. There are also additional domains that can “flexibly” assemble heterochromatin. These smaller domains are less enriched for H3K9me2 and can mark regions expressed in meiosis or sites of convergent gene expression and have been called heterochromatin islands [16]. A general schematic of proteins involved in heterochromatin assembly is shown in Figure 2.

### 3.1. ClrC Complex

In *S. pombe*, all H3 K9 methylation is catalyzed by a single methyltransferase, Clr4, which is a homolog of Suv39 [17]. Clr4 is a component of the ClrC complex, which, in addition to methyltransferase activity, has Cullin-4-dependent E3-ubiquitin ligase activity. The major histone target of ClrC ubiquitylation is H3K14, and H3K14ub is preferentially enriched in heterochromatin [18]. ClrC includes Rik1, Pcu4 (Cul4), Raf1 (Cmc1, Dos1, and Clr8), Raf2 (Cmc2, Dos2, and Clr7) and Rbx1 [19,20,21,22], and all components of ClrC are required for H3K9 methylation. The H3K9me2/3 mark in *S. pombe* is bound by the chromodomain-containing proteins Swi6 and Chp2 (homologs of HP1), Chp1, and by Clr4 itself [14,23,24,25], contributing to a positive feed-forward amplification and spreading mechanism as Clr4 not only reads H3K9me2/3 but “writes” it on adjacent nucleosomes. As an additional regulatory twist, Clr4 binds to H3K14ub tails, and the Ub mark promotes its methyltransferase activity [18]. Whether this interaction between H3K14ub and Clr4 promotes heterochromatin assembly at all or only some heterochromatic loci is currently a matter of debate [18,26].

ClrC complex recruitment to chromatin is central to heterochromatin assembly, and different mechanisms contribute to ClrC recruitment at distinct loci. For example, at centromeres, the RNAi pathway is central to heterochromatin maintenance, whereas at subtelomeres, RNAi contributes, but robust alternate mechanisms also recruit ClrC, so heterochromatin is unaffected by disruption of RNAi [27,28,29].

### 3.2. The RNAi Pathway

In *S. pombe*, the RNAi pathway contributes to both post-transcriptional gene silencing (where siRNA contributes to turn over of RNAs) and transcriptional gene silencing, with RNAi-directed recruitment of ClrC to assemble heterochromatin. Two important complexes of the RNAi machinery are RITS: RNA-induced initiation of transcriptional gene silencing, and RDRC (RNA-directed RNA polymerase complex) [29,30,31]. RITS includes the sole fission yeast Argonaute protein (Ago1), which, like mammalian Ago2, has siRNA-directed endonucleolytic or “slicer” activity [32]. Within RITS, the Tas3 (GW domain protein) links Ago1 to the chromodomain protein, Chp1, which binds H3K9me2/3 with high affinity [28,30,33,34]. RITS physically interacts with RDRC [35], which comprises RNA-dependent RNA polymerase (Rdp1), a putative RNA helicase (Hrr1), and polyA polymerase (Cid12) [30,35]. Briefly, low-level heterochromatic transcripts are converted to dsRNA by the activity of RDRC. Dicer physically associates with RDRC, then cleaves these dsRNAs to form short interfering RNAs (siRNAs), which are loaded into RITS. siRNA guides recruit RITS to homologous transcripts and RITS recruits ClrC, as well as cleaving nascent transcripts, contributing to heterochromatin assembly [29,30,32,34,35,36,37,38,39,40].

### 3.3. Role of Histone Deacetylases

Histone deacetylases (HDACs) remove acetyl groups from the ε-amino group of lysines within histone tails, a mark deposited by histone acetyltransferases (HATs) [41]. This is crucial in order to obtain the hypoacetylated state of heterochromatin and allow for methylation or other regulatory post-translational modifications. Deacetylation of H3K9 is catalyzed by Sir2, while Clr3 is the main HDAC responsible for deacetylation of histone H3 Lysine 14 (H3K14) [42]. Both HDACs are important in the maintenance of subtelomeric heterochromatin.

At the subtelomeric repeats, Sir2 HDAC activity is required for silencing [43,44,45] since the catalytically inactive mutant (Sir2 N247A) caused loss of telomeric silencing, with loss of H3K9me, increased H3K9ac, and loss of Swi6 localization [43,46].

Clr3 is a member of the Snf2/HDAC-containing repressor complex, SHREC, which can be recruited to heterochromatin via the Chp2 chromodomain protein binding H3K9me2/3. Clr3 was first identified in a genetic screen for mutants with disrupted mating type locus silencing [47] but also shows a partial defect in silencing at the outer repeats of the centromere and subtelomeric heterochromatin with no observed chromosome segregation defects [48,49,50]. In vitro data suggest the major target of Clr3 is H3K14ac along with some deacetylase activity towards H4K16ac [50]. Quantitative liquid chromatography–tandem mass spectrometry of purified histones (LC-MS/MS) revealed that Clr3 primarily targets H3K14ac on histones with H3K9me, indicating that it primarily operates at heterochromatic regions [51].

All components of SHREC are required for its role in heterochromatin maintenance at centromeres, mating type loci, and subtelomeres [52], and Clr3 and Mit1 both contribute enzymatic activities toward silencing, since Mit1 can mobilize nucleosomes onto regions that are refractory to nucleosome occupancy [53,54]. Interestingly, deacetylation of H3K14 does not appear to be the sole function of Clr3 since deletion of Clr3 has a more severe impact on nucleosome occupancy than seen in strains that prevent deposition of H3K14ac through the deletion of the responsible HATs. This indicates that Clr3 may serve additional roles along with the deacetylation of H3K14 [53]. Clr3 has been implicated in suppressing histone turnover at mating type and pericentromeric regions, preventing the dilution of heterochromatic histone PTMs and promoting the stable inheritance of heterochromatin [55]. The difference in phenotype between mutants in different SHREC subunits raises the possibility of a distinct role for different members of the complex, possibly acting through sub-complexes [56].

## 4. Subtelomeric DNA Sequences

**Figure 3:**
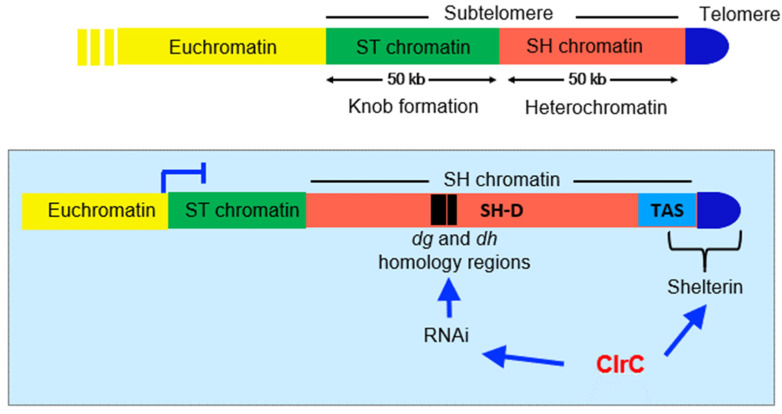
Subtelomeric domains in *S. pombe*, with a focus on SH domains, which consist of telomere-associated sequences (TAS) and SH-D domains on which heterochromatin assembles. Shelterin complex binds the telomere and TAS sequences and RNAi activity is directed through the *dg* and *dh* homology sequences resident within SH-D.

Subtelomeres in *S. pombe* span ~100 kb (Figure 3). They can be separated into two 50 kb domains: the subtelomere homologous domains (SH) which lie adjacent to the telomeric repeats, and the subtelomere unique domains (SU or ST, which we will hereon refer to as ST), which lie internal to the SH domains. Recent work from the Kanoh lab has revealed the exact sequences of the elusive portions of subtelomeric domains [6].

### 4.1. SH Domains

The SH domains of subtelomeres have high levels of sequence identity (>90% identity) between the ends of chromosomes 1 and 2. Even though the *S. pombe* genome sequence was published in 2002 [2], there remained uncertainty and gaps within the SH sequence assemblies which has slowed research on these domains. Many groups have contributed to the elucidation of chromosomal arrangement of subtelomeric sequences, including sequencing of a telomere plasmid library [3], sequence identification and mapping [57,58], and the heroic efforts of the Kanoh group who finally completed the subtelomeric sequence assemblies by mapping using their comprehensive series of strains bearing deletions of subtelomeric sequences [5,6]. These efforts have also highlighted the propensity for accumulation of mutations within SH sequences and suggest that these domains are a rich source of genetic variation. In man, these regions harbor several distinct repetitive elements, some of which contribute to open-reading frames, and are subject to mutation in disease, for example, the DUX4 homology genes at the termini of chromosomes 4 and 10, which are mutated in facioscapulohumeral muscular dystrophy, a muscle-wasting disease [59]. Subtelomeric domains are silenced, and loss of silencing can also lead to defects in subtelomere function and aberrant DUX4 expression, leading to pathogenesis [60,61]. The nature of telomeric and subtelomeric satellite sequences and their evolution has recently been extensively reviewed [62].

SH domains can be further subdivided into SH-D or distal regions, which lie distal to the telomere, and the much more highly variable SH-P or telomere-proximal domains- also known as TAS sequences that occupy 5–10 kb of sequence and directly abut the telomeric repeats.

#### 4.1.1. TAS Domains (SH-P)

TAS sequences are highly complex mosaic structures consisting of elements shared between the four subtelomeres but in mixed order and exhibiting nucleotide changes within the motifs. These characteristics indicate that these domains are hot spots for recombination and rearrangement [6]. TAS sequences are unusual with strong enrichment for poly [dA:dT] tracts, which have been shown to preclude nucleosome assembly [54]. TAS regions encode subtelomeric non-coding transcripts called TERRA, and a portion of these sequences, and possibly TERRA transcripts themselves, are included in telosome structures.

#### 4.1.2. SH-D Domains

SH-D domains show very high homology between the four ends of chromosomes 1 and 2, and remnants can be found between the telomeres and rDNA repeats on chromosome 3 in some strains. Due to the difficulty in sequence analysis and mapping, their exact sequence has remained ambiguous until very recently. Several genes lie within SH-D, including telomere-linked helicases, or *tlh* genes, all of which are non-essential. In the original sequence of the *S. pombe* genome, two *tlh* genes were assigned, but with the completion of sequence of subtelomeric domains, we now know that there are four of these very similar genes, one in each SH-D region [6]. Within the *tlh* gene sequences are elements known as *dg* and *dh* that are shared between sites of constitutive heterochromatin at centromeres, the mating type locus, and at subtelomeres [63]. These elements can initiate heterochromatin assembly through an RNAi-dependent pathway. Deletion of all SH-D regions within a strain is compatible with normal growth, and loss of SH-D does not affect telomerase recruitment [5]. However, in cells that lack telomerase, SH-D domains on chromosomes 1 and 2 play an important role in safeguarding cells by promoting intra-chromosomal circularization through single-stranded annealing between SH-D sequences shared between the left and right arms of the same chromosome [64]. In the absence of SH domains, cells lacking telomerase can undergo deleterious inter-chromosomal fusions. However, cells can survive such events if they undergo inter-chromosomal fusions through homologous sequences such as LTRs near the ends of the two chromosomes, followed by the inactivation of one of the centromeres [5].

### 4.2. ST Domains

ST domains comprise unique sequences that span approximately 50 kb and separate the euchromatic chromosomal domain from repetitive subtelomeric SH domains. Although the sequences of ST domains are not conserved across the subtelomeres of *S. pombe*, the assembly of a highly condensed chromatin “knob” is conserved on these domains [65].

## 5. Chromatin at Subtelomeric SH Domains

At subtelomeric regions of *S. pombe*, redundant mechanisms are responsible for the formation and maintenance of heterochromatin. As mentioned above, repetitive elements similar to the centromeric *dg* and *dh* repeats within SH-D can recruit ClrC via RNAi to form subtelomeric heterochromatin [63]. There are also RNAi-independent mechanisms of subtelomeric heterochromatin formation involving Taz1 and the shelterin complex, which can bind telomeric repeats and is required for silencing of SH-D domains and TAS domains also [27,66]. The binding of Taz1 at telomeric repeats allows for the recruitment of Clr4, which methylates H3K9, recruiting the chromodomain-containing protein Swi6. These two mechanisms are thought to act redundantly in the establishment of heterochromatin at subtelomeric repeats [27].

### 5.1. Linking Telomere Repeats to Subtelomeric Heterochromatin: Ccq1 and Shelterin

Shelterin performs multiple important functions including recruitment and activation of telomerase and recruitment of ClrC complex to telomeres and, through linking the single-stranded and double-stranded regions of the telomere, allows the spreading of ClrC into subtelomeric domains. Shelterin comprises the conserved subunits Pot1, Tpz1, Poz1, Rap1, and Taz1 and also includes Ccq1 in *S. pombe*. Taz1 (the ortholog of TRF1 and 2) binds directly to the double-stranded portion of the telomeric repeats [66,67], and Pot1 binds to the single-stranded G-tail [68]. Tpz1 binds Pot1, recruiting Tpz1 to the single-stranded telomere repeats. Taz1 links to Tpz1–Pot1 via Rap1 and Poz1, which bridge between these subcomplexes. Ccq1 associates with Tpz1 and is responsible for recruiting telomerase to maintain telomere repeat length [66,67,68,69,70,71]. Ccq1 also physically interacts with and recruits ClrC and SHREC to telomeric DNA repeats [52,72,73,74,75] (see Figure 2). Dissection of the interplay between Ccq1 and its binding partners the telomerase component Est1 and Clr3-SHREC suggests that Ccq1 interaction with these components is mutually exclusive and results in both positive and negative regulation of telomere length [71].

Telomeric repeats are sufficient to recruit shelterin and ClrC, and analysis of mutants that disrupt interactions between shelterin components reveals the importance of shelterin in not only recruiting ClrC but also allowing it to spread from telomeric repeats to subtelomeric TAS and SH-D sequences [72].

### 5.2. Unusual Chromatin Structure at TAS Domains

In contrast to other heterochromatic loci, the assembly of heterochromatin on TAS domains occurs independent of RNAi. TAS domains were recently shown to have very low nucleosome occupancy, with only about one third of the occupancy seen in euchromatic regions [74]. This at first seems counter-intuitive since repressed regions normally are enriched for repressive histone marks such as H3K9me2/3, and low nucleosome occupancy is refractory to heterochromatin spreading [54,55]. It is possible, however, that the low nucleosome density promotes access of repressive factors that suppress transcription within these domains (such as Ccq1). The low nucleosome occupancy is an intrinsic feature of the TAS sequence and is not linked to proximity to telomeric repeats since insertion of a partial TAS sequence into an euchromatic environment yielded the same nucleosome-depletion at the ectopic site [74]. This loss of nucleosomes is likely due to the AT-rich sequence at TAS sequences, which precludes nucleosome positioning [54,76]. Silencing of TAS sequences depends on the shelterin complex and the SHREC complex. SHREC includes the histone deacetylase Clr3 as well as the ATP-dependent Mit1 chromatin remodeler and has been shown to be important for moving nucleosomes onto sequences that are refractory for nucleosome positioning within the mating type region [53,54]; Ccq1 is necessary to maintain nucleosome occupancy at TAS as nucleosome density is further reduced in a *ccq1* mutant background [74]. Importantly, Ccq1 associates with and recruits both shelterin and SHREC complexes to TAS sequences, and in turn, shelterin is necessary for ClrC recruitment to telomeric sequences and subtelomeres. Silencing of chromatin at TAS sequences is also disrupted in *set2Δ* K36 methyltransferase mutants, leading to increased TERRA transcription. Deletion of the HAT complex Mst2-C also leads to upregulation of TERRA transcripts, but in contrast to sites of constitutive heterochromatin (SH-D domains- see below), the combination of *set2Δ* and *mst2Δ* does not suppress the silencing defect at TAS [77], suggesting that Set2 provides roles other than sequestration of Mst2 to silence TAS sequences.

### 5.3. Constitutive Heterochromatin at SH-D Domains

The chromatin that assembles on SH-D domains resembles constitutive heterochromatin, with high levels of H3K9me2/3. It is established by multiple factors, including the RNAi pathway, which acts through the *dg* and *dh* sequences in SH-D, and the telomere associated shelterin proteins, which reside at TAS and telomere repeats [16,27,72]. Heterochromatin spreads throughout the SH-D domain but appears to be limited from spreading into adjacent ST sequences. Mapping of heterochromatin at SH-D loci was not fully feasible until strains with just single SH-D domains were generated, due to the repetitive nature of the elements [5]. In strains that lack SH sequences, heterochromatin can now spread from the telomere through ~50 kb of SH-D adjacent sequences (ST) but is blocked by an apparent boundary that resides ~100 kb internal to the natural end of the chromosome that defines the border of euchromatin and subtelomeric chromatin and prevents further spread of heterochromatin [5]. At two out of the four subtelomeres, this boundary is a nucleosome-free region. Thus, there appear to be two mechanisms that limit the spreading of heterochromatin at subtelomeres. First, the SH sequence itself may block spreading, as deletion of SH facilitates spreading of heterochromatin through ST. This ability of heterochromatin to spread through ST may, however, simply be caused by the removal of ~50 kb of SH sequence, placing ST closer to telomere sequences. Second, the existence of a nucleosome-free region, at least at a subset of subtelomeres, effectively blocks the spreading of heterochromatin into euchromatin and prevents the silencing of essential genes. It is currently not clear how this nucleosome-depleted region is formed. What is known is that its formation occurs independent of the Fft3 chromatin remodeler, which suppresses euchromatic invasion of subtelomeric chromatin [78], and of the Shugoshin protein, Sgo2, which is important for condensation of ST chromatin [79].

#### Push Me–Pull You between Distinct Activatory/Repressive Complexes

Whilst heterochromatic loci are associated with low levels of activatory chromatin marks, intriguingly, the loss of the enzymes that generate the activatory marks H3K4me3 and H3K36me3 leads to derepression of particular sites of heterochromatin. The loss of Set2 (K36 methylation) leads to derepression of all sites of constitutive heterochromatin and the loss of Set1 (K4 methylation) to defective heterochromatin at subtelomeres and heterochromatic islands. Both enzymes associate with the C-terminal domain of RNA Pol II and mark sites of transcriptional activity in euchromatin. In both instances, loss of the methylated histone products leads to deregulation of either the localization or the activity of different HAT complexes, leading to aberrant transcription of normally heterochromatic sites. The loss of the components of Set1-C results in the loss of H3K4 methylation and a loss of antagonism with the H3K4 histone acetyltransferase Mst1. In cells lacking Set1-C, Mst1 can then acetylate and derepress subtelomeric heterochromatin [80]. In contrast, K36 tri-methylation recruits the Mst2-C histone acetyltransferase complex to euchromatin. In cells lacking Set2 or the subunit of Mst2-C that recruits it to H3K36me3, Mst2 is no longer tethered to euchromatin and is free to derepress sites of constitutive heterochromatin [77,81]. In keeping with this, the double deletion of both Set2 and Mst2 suppresses the loss of heterochromatin in *set2Δ* alone. These examples highlight the importance of proper balance between chromatin modifiers in control of subtelomeric heterochromatin. Recent work has shed further light on the mechanism of Mst2 function. Within euchromatin, Mst2 acetylates the Brl1 ubiquitin ligase at K242, which is required for the activation of its ubiquitin ligase activity on H2BK119. According to Flury et al. [81], euchromatin is maintained by a feedback loop consisting of RNA Pol II, Set2, Mst2, Brl1, and H2B-K119ub. RNA Pol II recruits Set2 to methylate H3K36, and H3K36me3 then attracts Mst2-C. Mst2 acetylates Brl1, and acetylated Brl1 ubiquitinates H2B-K119, and finally, H2B-K119ub recruits RNA Pol II, coming the full circle. It has been proposed that the major role of Set2 and K36 methylation is to sequester Mst2 and restrain H2B-K119ub to actively transcribed domains and to limit access to heterochromatin [81]. However, while the loss of Mst2 compensates for the silencing defect of *set2Δ* at heterochromatic sites, the combination of *set2Δ* with the non-acetylatable Brl1 K242R mutant does not compensate for *set2Δ*’s heterochromatin silencing defect, suggesting that the role of Mst2 in counteracting heterochromatic silencing is independent of its role in H2B ubiquitin regulation [77].

## 6. Specialized Chromatin and Knob Formation on ST Domains

The ST regions adjacent to the subtelomeric SH silent chromatin are distinguished by their unique chromatin features. These regions are characterized by very low levels of histone variant H2A.Z and histone H3 lysine methylation at K4, K9, and K36 [65,82]. The ST regions are the sites at which a ‘neocentromere’ can form after an authentic centromere is lost [83]. Furthermore, the ST chromatin regions form a cluster of highly condensed chromatin domains, called knobs, in the interphase nucleus [65]. The knobs disappear during mitosis and re-form in the subsequent G1 phase [65].

### 6.1. Histone H3K36 Epigenetic States Are Critical for Knob Formation

**Figure 4:**
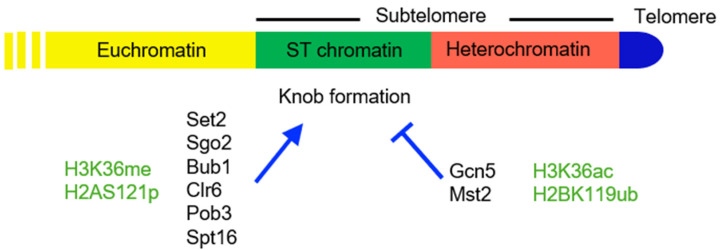
The ST domain in *S. pombe.* Protein factors (black) and histone modifications (green) that promote (arrowhead) or inhibit (blunt arrow) knob formation are shown below the map.

Histone H3 lysine 36 (H3-K36) plays an important regulatory role in the formation of knobs on the ST chromatin (Figure 4). H3K36 can be both methylated and acetylated, and both modifications play a crucial role in knob formation. Set2 is the sole methyltransferase for H3K36 in *S. pombe* [84] and elicits mono-, di-, and tri-methylation of K36. Set2 associates with the C-terminus of RNA Pol II, so K36 methylation is a consequence of RNA Pol II passage [85] but can also function to suppress spurious transcription from cryptic promoters through recruitment of HDAC activity [86,87]. Deletion of the *set2* gene (*set2∆*) results in complete loss of K36 methylation and almost completely blocks knob formation [65], suggesting that methylation of H3K36 promotes knob formation. Replacement of H3K36 with another basic residue, H3-K36R, prevents both methylation and acetylation of H3K36 and decreases knob formation, but not to the extent seen in *set2Δ* [65] (note that strains that retain a single histone H3 gene where mutations are engineered into the sole copy histone are designated H3-K36R etc. (see Yadav et al. for details [88])). This result may suggest that Set2-mediated methylation of another non-histone protein target is important for knob formation, or conversely that knob formation is also influenced by a non-enzymatic role of Set2, such as a potential scaffolding function, perhaps linking other regulatory proteins to RNA Pol II via association with Set2. Intriguingly, H3-K36Q mutation, which also prevents K36 methylation but mimics H3K36 acetylation, completely eliminates knob formation [65], suggesting that acetylation of histone H3K36 is sufficient to decondense the knobs at the ST chromatin. Together, these data support a model wherein low levels of H3K36 acetylation and high levels of H3K36 methylation are required for knob formation.

Further evidence supporting the involvement of acetylation status of H3K36 in knob formation came from study of a nearby residue in histone H3, H3-G34, which is mutated in pediatric high-grade glioma [88,89,90,91]. Both histone H3-G34R and H3-G34V mutations led to reduced H3K36me3, accumulation of H3K36me2, and transcriptional repression of ST domains. Further, H3-G34R also prevented H3K36 acetylation [88] and caused an increase in knob formation [91], whereas H3-G34V did not affect either H3K36 acetylation or knob formation [91]. These data support the hypothesis that histone H3-G34R induces knob formation through reducing H3K36 acetylation levels.

Yet further support for a regulatory role of histone acetylation in knob formation is that a double mutant of histone acetyl transferases *gcn5∆* and *mst2∆* reduces H3K36ac, represses transcription of ST domains, and concomitantly increases knob formation [91]. Mst2 is known to be recruited to H3K36me3-marked chromatin through its association with the PWWP domain protein Pdp3 and to acetylate not only histones but also specifically K242 of the E3 ubiquitin protein ligase Brl1 [81]. Brl1, in turn, is the E3 ubiquitin ligase enzyme for K119 of histone H2B, and its acetylation by Mst2 is required for ubiquitination of H2B [81,92,93]. This ubiquitination at H2B-K119 is associated with transcriptional activation and is known to prevent knob formation [94]. Intriguingly, in contrast to the heterochromatic SH subtelomeric domain, where the loss of Mst2 or Set2 causes transcript accumulation and the combination of *set2∆* and *mst2∆* results in proper silencing, transcripts within knob regions are upregulated in *set2∆* but repressed in *mst2∆*, but the combined mutation of *set2∆* and *mst2∆* is not sufficient to restore silencing defect of a *set2∆* strain.

As mentioned earlier, Mst2 and Set2 are involved in a feedback loop to maintain euchromatin. Among these elements in the feedback loop, Set2 promotes while Mst2 and H2BK119ub inhibit knob formation. At a first glance, the opposing roles of Set2 and of Mst2 and H2BK119ub seem inconsistent. However, Mst2 acetylates H3K36 [91], and it has been reported that cells lacking Set2 have elevated levels of H3K36ac [95], although this was not seen in another study [91]. Alternately, because levels of H3K36me2 and H3K36me3 are low at the knob region [65], only H3K36me1 may be necessary for knob formation at the ST chromatin.

### 6.2. Sgo2 Localized at the Subtelomeric Regions Promotes Knob Formation

Shugoshin protein Sgo2 is localized at the centromere and contributes to precise chromosome segregation during mitosis [96,97]. During the interphase, Sgo2 re-localizes to the subtelomeric regions and promotes knob formation [79]. Deletion mutants of *sgo2∆* completely lose knobs [79], indicating that Sgo2 plays a major role in chromatin condensation at ST-chromatin, though the mechanism of chromatin condensation is not yet known. What is clear is that in *set2Δ* mutants, the amount of Sgo2 localized at ST is decreased [79]. Conversely, in histone H3-G34R mutants and in the double acetyltransferase mutant (*mst2Δ gcn5Δ*), there is increased Sgo2 localization at the subtelomeric regions, whereas in H3-G34V mutants, there is not [91], consistent with the premise that H3-G34R induces knob formation through reducing H3K36 acetylation levels. These data indicate that Sgo2 localization is downstream of histone H3K36 modification.

**Figure 5:**
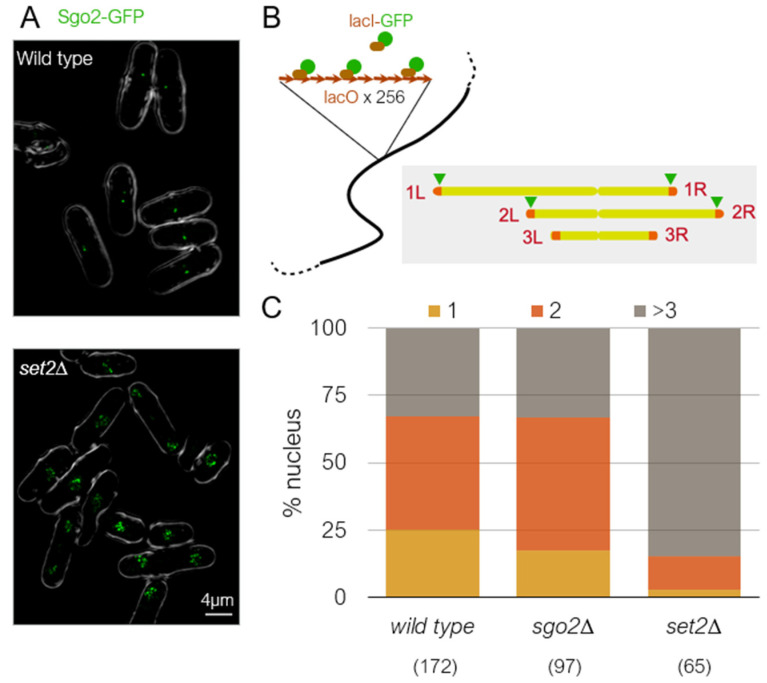
Factors regulating subtelomeric clustering and knob formation. (**A**) Sgo2-GFP localization in wild-type and *set2∆* cells. Sgo2-GFP localizes to the subtelomeric regions of chromosomes 1 and 2 in interphase. The Sgo2-GFP foci cluster to make a single dot in wild-type cells but are dispersed in *set2∆* cells. (**B**) The LacO–LacI system to visualize chromosomal loci. The *lacO* array was inserted near the four boundaries between euchromatin and ST chromatin of chromosomes 1 and 2 (AC1, AC26, CII-L, and CII-R in Matsuda et al., 2015 [65]) and visualized by lacI-GFP. (**C**) Number of apparent lacI-GFP dots in wild-type and mutant nuclei. While many nuclei contained only 1 or 2 dots in wild-type and *sgo2∆*, the majority of nuclei contained more than 3 dots in *set2∆* nuclei, indicating that subtelomeric regions are not clustered in *set2∆*. Numbers in parentheses indicate number of cells examined.

Subtelomeric regions are known to physically cluster in the nucleus in many eukaryotes [98,99,100]. Although the molecular mechanism for this clustering is unknown, silencing factors are known to be required for the long-range subtelomeric interaction in budding yeast [101,102]. In fission yeast, re-examination of genome-wide Hi-C data [103] revealed that subtelomeres are clustered not through the SH domain, but through the ST-chromatin region [65]. Consistently, the subtelomeric regions of only chromsomes 1 and 2 are clustered, while chromosome 3 is not [65,103]. Therefore, it is interesting to know whether subtelomeric clustering in *S. pombe* is related to condensed knob formation. RNAi mutants, *ago1∆*, *dcr1∆,* and *rdp1∆*, are defective in subtelomeric clustering in *S. pombe* [104]. In *dcr1∆* cells, knob formation was only slightly reduced [65]. This may mean that subtelomeric clustering is not required for knob formation, or the remaining capability to form subtelomeric foci in *dcr1∆* is sufficient for knob formation. We found that clustering of the subtelomeric regions requires Set2 (Figure 5A), indicating that chromatin clustering requires epigenetic marks at H3K36 similar to knob condensation. To examine whether Sgo2 is required for clustering, we created a strain harboring lacO repeat arrays inserted near the four boundaries between euchromatin and ST chromatin and visualized by LacI-GFP (Figure 5B). Interestingly, clustering of the subtelomeric regions still occurs in the absence of Sgo2 (Figure 5C). Therefore, subtelomeric clustering and chromatin condensation are separate processes in *S. pombe*. Furthermore, because gene expression levels in subtelomeric regions are similarly elevated in *set2∆* and *sgo2∆* [65,79], the fact that subtelomeres still cluster in *sgo2∆* indicates that clustering occurs independently of gene silencing at the subtelomeres.

## References

[1] Kwapisz M., Morillon A. (2020). Subtelomeric Transcription and its Regulation. J. Mol. Biol..

[2] Wood V., Gwilliam R., Rajandream M.-A., Lyne M., Lyne R., Stewart A., Sgouros J., Peat N., Hayles J., Baker S. (2002). The genome sequence of Schizosaccharomyces pombe. Nat. Cell Biol..

[3] Sugawara N. (1988). DNA Sequences at the Telomeres of the Fission Yeast Schizosaccharomyces Pombe. Ph.D. Thesis.

[4] Hiraoka Y., Henderson E., Blackburn E.H. (1998). Not so peculiar: Fission yeast telomere repeats. Trends Biochem. Sci..

[5] Tashiro S., Nishihara Y., Kugou K., Ohta K., Kanoh J. (2017). Subtelomeres constitute a safeguard for gene expression and chromosome homeostasis. Nucleic Acids Res..

[6] Oizumi Y., Kaji T., Tashiro S., Takeshita Y., Date Y., Kanoh J. (2021). Complete sequences of Schizosaccharomyces pombe subtelomeres reveal multiple patterns of genome variation. Nat. Commun..

[7] Hirano Y., Asakawa H., Sakuno T., Haraguchi T., Hiraoka Y. (2020). Nuclear Envelope Proteins Modulating the Heterochromatin Formation and Functions in Fission Yeast. Cells.

[8] Chikashige Y., Yamane M., Okamasa K., Tsutsumi C., Kojidani T., Sato M., Haraguchi T., Hiraoka Y. (2009). Membrane proteins Bqt3 and -4 anchor telomeres to the nuclear envelope to ensure chromosomal bouquet formation. J. Cell Biol..

[9] Maestroni L., Reyes C., Vaurs M., Gachet Y., Tournier S., Géli V., Coulon S. (2020). Nuclear envelope attachment of telomeres limits TERRA and telomeric rearrangements in quiescent fission yeast cells. Nucleic Acids Res..

[10] Steglich B., Strålfors A., Khorosjutina O., Persson J., Smialowska A., Javerzat J.-P., Ekwall K. (2015). The Fun30 Chromatin Remodeler Fft3 Controls Nuclear Organization and Chromatin Structure of Insulators and Subtelomeres in Fission Yeast. PLoS Genet..

[11] Greenwood J., Patel H., Cech T.R., Cooper J.P. (2018). Fission yeast telosomes: Non-canonical histone-containing chromatin structures dependent on shelterin and RNA. Nucleic Acids Res..

[12] Luger K., Mäder A.W., Richmond R.K., Sargent D.F., Richmond T.J. (1997). Crystal structure of the nucleosome core particle at 2.8 Å resolution. Nature.

[13] Strahl B.D., Allis C.D. (2000). The language of covalent histone modifications. Nat. Cell Biol..

[14] Allis C.D., Jenuwein T. (2016). The molecular hallmarks of epigenetic control. Nat. Rev. Genet..

[15] Nakayama J.-I., Rice J.C., Strahl B.D., Allis C.D., Grewal S.I.S. (2001). Role of Histone H3 Lysine 9 Methylation in Epigenetic Control of Heterochromatin Assembly. Science.

[16] Cam H.P., Sugiyama T., Chen E.S., Chen X., FitzGerald P.C., Grewal S.I.S. (2005). Comprehensive analysis of heterochromatin- and RNAi-mediated epigenetic control of the fission yeast genome. Nat. Genet..

[17] Rea S., Eisenhaber F., O’Carroll D., Strahl B.D., Sun Z.-W., Schmid M., Opravil S., Mechtler K., Ponting C.P., Allis C.D. (2000). Regulation of chromatin structure by site-specific histone H3 methyltransferases. Nature.

[18] Oya E., Nakagawa R., Yoshimura Y., Tanaka M., Nishibuchi G., Machida S., Shirai A., Ekwall K., Kurumizaka H., Tagami H. (2019). H3K14 ubiquitylation promotes H3K9 methylation for heterochromatin assembly. EMBO Rep..

[19] Horn P.J., Bastie J.-N., Peterson C.L. (2005). A Rik1-associated, cullin-dependent E3 ubiquitin ligase is essential for heterochromatin formation. Genes Dev..

[20] Hong E.-J.E., Villen J., Moazed D. (2005). A Cullin E3 Ubiquitin Ligase Complex Associates with Rik1 and the Clr4 Histone H3-K9 Methyltransferase and is Required for RNAi-Mediated Heterochromatin Formation. RNA Biol..

[21] Jia S., Kobayashi R., Grewal S.I.S. (2005). Ubiquitin ligase component Cul4 associates with Clr4 histone methyltransferase to assemble heterochromatin. Nat. Cell Biol..

[22] Li F., Goto D.B., Zaratiegui M., Tang X., Martienssen R., Cande W.Z. (2005). Two Novel Proteins, Dos1 and Dos2, Interact with Rik1 to Regulate Heterochromatic RNA Interference and Histone Modification. Curr. Biol..

[23] Bannister A., Zegerman P., Partridge J., Miska E., Thomas J.O., Allshire R., Kouzarides T. (2001). Selective recognition of methylated lysine 9 on histone H3 by the HP1 chromo domain. Nat. Cell Biol..

[24] Partridge J., Scott K.S., Bannister A., Kouzarides T., Allshire R.C. (2002). cis-Acting DNA from Fission Yeast Centromeres Mediates Histone H3 Methylation and Recruitment of Silencing Factors and Cohesin to an Ectopic Site. Curr. Biol..

[25] Zhang K., Mosch K., Fischle W., Grewal S. (2008). Roles of the Clr4 methyltransferase complex in nucleation, spreading and maintenance of heterochromatin. Nat. Struct. Mol. Biol..

[26] Stirpe A., Guidotti N.J., Northall S.J., Kilic S., Hainard A., Vadas O., Fierz B., Schalch T. (2021). SUV39 SET domains mediate crosstalk of heterochromatic histone marks. BioRxiv.

[27] Kanoh J., Sadaie M., Urano T., Ishikawa F. (2005). Telomere Binding Protein Taz1 Establishes Swi6 Heterochromatin Independently of RNAi at Telomeres. Curr. Biol..

[28] Petrie V.J., Wuitschick J.D., Givens C.D., Kosinski A.M., Partridge J.F. (2005). RNA Interference (RNAi)-Dependent and RNAi-Independent Association of the Chp1 Chromodomain Protein with Distinct Heterochromatic Loci in Fission Yeast. Mol. Cell. Biol..

[29] Volpe T.A., Kidner C., Hall I.M., Teng G., Grewal S.I.S., Martienssen R.A. (2002). Regulation of Heterochromatic Silencing and Histone H3 Lysine-9 Methylation by RNAi. Science.

[30] Verdel A., Jia S., Gerber S., Sugiyama T., Gygi S., Grewal S.I.S., Moazed D. (2004). RNAi-Mediated Targeting of Heterochromatin by the RITS Complex. Science.

[31] Martienssen A.R., Moazed D. (2015). RNAi and Heterochromatin Assembly. Cold Spring Harb. Perspect. Biol..

[32] Irvine D.V., Zaratiegui M., Tolia N.H., Goto D.B., Chitwood D.H., Vaughn M.W., Joshua-Tor L., Martienssen R.A. (2006). Argonaute Slicing Is Required for Heterochromatic Silencing and Spreading. Science.

[33] DeBeauchamp J.L., Moses A., Noffsinger V.J.P., Ulrich D.L., Job G., Kosinski A.M., Partridge J.F. (2008). Chp1-Tas3 Interaction Is Required to Recruit RITS to Fission Yeast Centromeres and for Maintenance of Centromeric Heterochromatin. Mol. Cell. Biol..

[34] Schalch T., Job G., Noffsinger V.J., Shanker S., Kuscu C., Joshua-Tor L., Partridge J.F. (2009). High-Affinity Binding of Chp1 Chromodomain to K9 Methylated Histone H3 Is Required to Establish Centromeric Heterochromatin. Mol. Cell.

[35] Motamedi M.R., Verdel A., Colmenares S.U., Gerber S., Gygi S.P., Moazed D. (2004). Two RNAi Complexes, RITS and RDRC, Physically Interact and Localize to Noncoding Centromeric RNAs. Cell.

[36] Verdel A., Moazed D. (2005). RNAi-directed assembly of heterochromatin in fission yeast. FEBS Lett..

[37] Djupedal I., Portoso M., Spåhr H., Bonilla C., Gustafsson C.M., Allshire R., Ekwall K. (2005). RNA Pol II subunit Rpb7 promotes centromeric transcription and RNAi-directed chromatin silencing. Genes Dev..

[38] Kato H., Goto D.B., Martienssen R.A., Urano T., Furukawa K., Murakami Y. (2005). RNA Polymerase II Is Required for RNAi-Dependent Heterochromatin Assembly. Science.

[39] Creamer K.M., Partridge J.F. (2011). RITS-connecting transcription, RNA interference, and heterochromatin assembly in fission yeast. Wiley Interdiscip. Rev. RNA.

[40] Allshire R., Madhani H.D. (2018). Ten principles of heterochromatin formation and function. Nat. Rev. Mol. Cell Biol..

[41] Seto E., Yoshida M. (2014). Erasers of Histone Acetylation: The Histone Deacetylase Enzymes. Cold Spring Harb. Perspect. Biol..

[42] Wirén M., Silverstein A.R., Sinha I., Walfridsson J., Lee H.-M., Laurenson P., Pillus L., Robyr D., Grunstein M., Ekwall K. (2005). Genomewide analysis of nucleosome density histone acetylation and HDAC function in fission yeast. EMBO J..

[43] Alper B.J., Job G., Yadav R., Shanker S., Lowe B., Partridge J. (2013). Sir2 is required for Clr4 to initiate centromeric heterochromatin assembly in fission yeast. EMBO J..

[44] Freeman-Cook L.L., Gómez E.B., Spedale E.J., Marlett J., Forsburg S., Pillus L., Laurenson P. (2005). Conserved Locus-Specific Silencing Functions of Schizosaccharomyces pombe sir2+. Genetics.

[45] Shankaranarayana G.D., Motamedi M.R., Moazed D., Grewal S.I. (2003). Sir2 Regulates Histone H3 Lysine 9 Methylation and Heterochromatin Assembly in Fission Yeast. Curr. Biol..

[46] Min J., Landry J., Sternglanz R., Xu R.-M. (2001). Crystal Structure of a SIR2 Homolog–NAD Complex. Cell.

[47] Ekwall K., Ruusala T. (1994). Mutations in rik1, clr2, clr3 and clr4 genes asymmetrically derepress the silent mating-type loci in fission yeast. Genetics.

[48] Allshire R., Nimmo E.R., Ekwall K., Javerzat J.P., Cranston G. (1995). Mutations derepressing silent centromeric domains in fission yeast disrupt chromosome segregation. Genes Dev..

[49] Ekwall K., Nimmo E.R., Javerzat J.P., Borgstrom B., Egel R., Cranston G., Allshire R. (1996). Mutations in the fission yeast silencing factors clr4+ and rik1+ disrupt the localisation of the chromo domain protein Swi6p and impair centromere function. J. Cell Sci.

[50] Bjerling P., Silverstein R.A., Thon G., Caudy A., Grewal S., Ekwall K. (2002). Functional Divergence between Histone Deacetylases in Fission Yeast by Distinct Cellular Localization and In Vivo Specificity. Mol. Cell. Biol..

[51] Abshiru N., Rajan R.E., Verreault A., Thibault P. (2016). Unraveling Site-Specific and Combinatorial Histone Modifications Using High-Resolution Mass Spectrometry in Histone Deacetylase Mutants of Fission Yeast. J. Proteome Res..

[52] Sugiyama T., Cam H.P., Sugiyama R., Noma K.-I., Zofall M., Kobayashi R., Grewal S.I. (2007). SHREC, an Effector Complex for Heterochromatic Transcriptional Silencing. Cell.

[53] Creamer K.M., Job G., Shanker S., Neale G.A., Lin Y.-C., Bartholomew B., Partridge J.F. (2014). The Mi-2 Homolog Mit1 Actively Positions Nucleosomes within Heterochromatin to Suppress Transcription. Mol. Cell. Biol..

[54] Garcia J., Dumesic P.A., Hartley P.D., El-Samad H., Madhani H.D. (2010). Combinatorial, site-specific requirement for heterochromatic silencing factors in the elimination of nucleosome-free regions. Genes Dev..

[55] Aygün O., Mehta S., Grewal S.I.S. (2013). HDAC-mediated suppression of histone turnover promotes epigenetic stability of heterochromatin. Nat. Struct. Mol. Biol..

[56] Job G., Brugger C., Xu T., Lowe B., Pfister Y., Qu C., Shanker S., Sanz J.I.B., Partridge J.F., Schalch T. (2016). SHREC Silences Heterochromatin via Distinct Remodeling and Deacetylation Modules. Mol. Cell.

[57] Chaudari A., Huberman A.J. (2012). Identification of two telomere-proximal fission yeast DNA replication origins constrained by nearby cis-acting sequences to replicate in late S phase. F1000Research.

[58] Van Emden T.S., Braun S. (2019). TASks for subtelomeres: When nucleosome loss and genome instability are favored. Curr. Genet..

[59] Lemmers R.J., Wohlgemuth M., van der Gaag K.J., van der Vliet P.J., van Teijlingen C.M., de Knijff P., Padberg G.W., Frants R.R., van der Maarel S.M. (2007). Specific Sequence Variations within the 4q35 Region Are Associated with Facioscapulohumeral Muscular Dystrophy. Am. J. Hum. Genet..

[60] Van Overveld P.G.M., Lemmers R.J.F.L., Sandkuijl A.L., Enthoven L., Winokur S.T., Bakels F., Padberg G.W., Van Ommen G.-J.B., Frants R.R., van der Maarel S. (2003). Hypomethylation of D4Z4 in 4q-linked and non-4q-linked facioscapulohumeral muscular dystrophy. Nat. Genet..

[61] Zeng W., de Greef J., Chen Y.-Y., Chien R., Kong X., Gregson H.C., Winokur S.T., Pyle A., Robertson K., Schmiesing J.A. (2009). Specific Loss of Histone H3 Lysine 9 Trimethylation and HP1γ/Cohesin Binding at D4Z4 Repeats Is Associated with Facioscapulohumeral Dystrophy (FSHD). PLoS Genet..

[62] Thakur J., Packiaraj J., Henikoff S. (2021). Sequence, Chromatin and Evolution of Satellite DNA. Int. J. Mol. Sci..

[63] Hansen K.R., Ibarra P.T., Thon G. (2006). Evolutionary-conserved telomere-linked helicase genes of fission yeast are repressed by silencing factors, RNAi components and the telomere-binding protein Taz1. Nucleic Acids Res..

[64] Wang X., Baumann P. (2008). Chromosome Fusions following Telomere Loss Are Mediated by Single-Strand Annealing. Mol. Cell.

[65] Matsuda A., Chikashige Y., Ding D.-Q., Ohtsuki C., Mori C., Asakawa H., Kimura H., Haraguchi T., Hiraoka Y. (2015). Highly condensed chromatins are formed adjacent to subtelomeric and decondensed silent chromatin in fission yeast. Nat. Commun..

[66] Cooper J.P., Nimmo E.R., Allshire R., Cech T.R. (1997). Regulation of telomere length and function by a Myb-domain protein in fission yeast. Nat. Cell Biol..

[67] Cooper J.P., Watanabe Y., Nurse P. (1998). Fission yeast Taz1 protein is required for meiotic telomere clustering and recombination. Nat. Cell Biol..

[68] Baumann P., Cech T.R. (2001). Pot1, the Putative Telomere End-Binding Protein in Fission Yeast and Humans. Science.

[69] Miyoshi T., Kanoh J., Saito M., Ishikawa F. (2008). Fission Yeast Pot1-Tpp1 Protects Telomeres and Regulates Telomere Length. Science.

[70] Tomita K., Cooper J.P. (2008). Fission yeast Ccq1 is telomerase recruiter and local checkpoint controller. Genes Dev..

[71] Armstrong C.A., Moiseeva V., Collopy L.C., Pearson S.R., Ullah T.R., Xi S.T., Martin J., Subramaniam S., Marelli S., Amelina H. (2017). Fission yeast Ccq1 is a modulator of telomerase activity. Nucleic Acids Res..

[72] Wang J., Cohen A.L., Letian A., Tadeo X., Moresco J.J., Liu J., Yates J.R., Qiao F., Jia S. (2016). The proper connection between shelterin components is required for telomeric heterochromatin assembly. Genes Dev..

[73] Zofall M., Smith D.R., Mizuguchi T., Dhakshnamoorthy J., Grewal S.I. (2016). Taz1-Shelterin Promotes Facultative Heterochromatin Assembly at Chromosome-Internal Sites Containing Late Replication Origins. Mol. Cell.

[74] van Emden T.S., Forn M., Forné I., Sarkadi Z., Capella M., Caballero L.M., Fischer-Burkart S., Brönner C., Simonetta M., Toczyski D. (2019). Shelterin and subtelomeric DNA sequences control nucleosome maintenance and genome stability. EMBO Rep..

[75] Moser B.A., Raguimova O.N., Nakamura T.M. (2015). Ccq1-Tpz1TPP1 interaction facilitates telomerase and SHREC association with telomeres in fission yeast. Mol. Biol. Cell.

[76] Xi L., Fondufe-Mittendorf Y., Xia L., Flatow J., Widom J., Wang J.-P. (2010). Predicting nucleosome positioning using a duration Hidden Markov Model. BMC Bioinform..

[77] Georgescu P., Capella M., Fischer-Burkart S., Braun S. (2020). The euchromatic histone mark H3K36me3 preserves heterochromatin through sequestration of an acetyltransferase complex in fission yeast. Microb. Cell.

[78] Strålfors A., Walfridsson J., Bhuiyan H., Ekwall K. (2011). The FUN30 Chromatin Remodeler, Fft3, Protects Centromeric and Subtelomeric Domains from Euchromatin Formation. PLoS Genet..

[79] Tashiro S., Handa T., Matsuda A., Ban T., Takigawa T., Miyasato K., Ishii K., Kugou K., Ohta K., Hiraoka Y. (2016). Shugoshin forms a specialized chromatin domain at subtelomeres that regulates transcription and replication timing. Nat. Commun..

[80] Mikheyeva I.V., Grady P.J.R., Tamburini F.B., Lorenz D.R., Cam H.P. (2014). Multifaceted Genome Control by Set1 Dependent and Independent of H3K4 Methylation and the Set1C/COMPASS Complex. PLoS Genet..

[81] Flury V., Georgescu P., Iesmantavicius V., Shimada Y., Kuzdere T., Braun S., Bühler M. (2017). The Histone Acetyltransferase Mst2 Protects Active Chromatin from Epigenetic Silencing by Acetylating the Ubiquitin Ligase Brl1. Mol. Cell.

[82] Buchanan L., Durand-Dubief M., Roguev A., Sakalar C., Wilhelm B., Strålfors A., Shevchenko A., Aasland R., Shevchenko A., Ekwall K. (2009). The Schizosaccharomyces pombe JmjC-Protein, Msc1, Prevents H2A.Z Localization in Centromeric and Subtelomeric Chromatin Domains. PLoS Genet..

[83] Ishii K., Ogiyama Y., Chikashige Y., Soejima S., Masuda F., Kakuma T., Hiraoka Y., Takahashi K. (2008). Heterochromatin Integrity Affects Chromosome Reorganization After Centromere Dysfunction. Science.

[84] Morris S.A., Shibata Y., Noma K.-I., Tsukamoto Y., Warren E., Temple B., Grewal S.I.S., Strahl B.D. (2005). Histone H3 K36 Methylation Is Associated with Transcription Elongation in Schizosaccharomyces pombe. Eukaryot. Cell.

[85] Wagner E.J., Carpenter P.B. (2012). Understanding the language of Lys36 methylation at histone H3. Nat. Rev. Mol. Cell Biol..

[86] Carrozza M.J., Li B., Florens L., Suganuma T., Swanson S.K., Lee K.K., Shia W.-J., Anderson S., Yates J., Washburn M. (2005). Histone H3 Methylation by Set2 Directs Deacetylation of Coding Regions by Rpd3S to Suppress Spurious Intragenic Transcription. Cell.

[87] Nicolas E., Yamada T., Cam H.P., Fitzgerald P.C., Kobayashi R., Grewal S. (2007). Distinct roles of HDAC complexes in promoter silencing, antisense suppression and DNA damage protection. Nat. Struct. Mol. Biol..

[88] Wu G., Broniscer A., McEachron T.A., Lu C., Paugh B.S., Becksfort J., Qu C., Ding L., Huether R., Parker M. (2012). Somatic histone H3 alterations in pediatric diffuse intrinsic pontine gliomas and non-brainstem glioblastomas. Nat. Genet..

[89] Schwartzentruber J., Korshunov A., Liu X.-Y., Jones D.T.W., Pfaff E., Jacob K., Sturm D., Fontebasso A.M., Khuong-Quang D.-A., Tönjes M. (2012). Driver mutations in histone H3.3 and chromatin remodelling genes in paediatric glioblastoma. Nature.

[90] Yadav R., Jablonowski C.M., Fernandez A.G., Lowe B.R., Henry A.R., Finkelstein D., Barnum K.J., Pidoux A.L., Kuo Y.-M., Huang J. (2017). Histone H3G34R mutation causes replication stress, homologous recombination defects and genomic instability in S. pombe. eLife.

[91] Lowe B.R., Yadav R.K., Henry A.R., Schreiner P., Matsuda A., Fernandez A.G., Finkelstein D., Campbell M., Kallappagoudar S., Jablonowski C.M. (2021). Surprising phenotypic diversity of cancer-associated mutations of Gly 34 in the histone H3 tail. eLife.

[92] Tanny J.C., Erdjument-Bromage H., Tempst P., Allis C.D. (2007). Ubiquitylation of histone H2B controls RNA polymerase II transcription elongation independently of histone H3 methylation. Genes Dev..

[93] Zofall M., Grewal S.I. (2007). HULC, a Histone H2B Ubiquitinating Complex, Modulates Heterochromatin Independent of Histone Methylation in Fission Yeast. J. Biol. Chem..

[94] Murawska M., Schauer T., Matsuda A., Wilson M., Pysik T., Wojcik F., Muir T.W., Hiraoka Y., Straub T., Ladurner A.G. (2020). The Chaperone FACT and Histone H2B Ubiquitination Maintain S. pombe Genome Architecture through Genic and Subtelomeric Functions. Mol. Cell.

[95] Pai C.-C., Deegan R.S., Subramanian L., Gal C., Sarkar S., Blaikley E.J., Walker C., Hulme L., Bernhard E., Codlin S. (2014). A histone H3K36 chromatin switch coordinates DNA double-strand break repair pathway choice. Nat. Commun..

[96] Kawashima S.A., Yamagishi Y., Honda T., Ishiguro K.-I., Watanabe Y. (2010). Phosphorylation of H2A by Bub1 Prevents Chromosomal Instability Through Localizing Shugoshin. Science.

[97] Vanoosthuyse V., Prykhozhij S., Hardwick K.G. (2007). Shugoshin 2 Regulates Localization of the Chromosomal Passenger Proteins in Fission Yeast Mitosis. Mol. Biol. Cell.

[98] Wesolowska N., Amariei F.L., Rong Y.S. (2013). Clustering and Protein Dynamics of Drosophila melanogaster Telomeres. Genetics.

[99] Crabbe L., Cesare A.J., Kasuboski J.M., Fitzpatrick J.A., Karlseder J. (2012). Human Telomeres Are Tethered to the Nuclear Envelope during Postmitotic Nuclear Assembly. Cell Rep..

[100] Freitas-Junior L.H., Bottius E., Pirrit L.A., Deitsch K.W., Scheidig C., Guinet F., Nehrbass U., Wellems T.E., Scherf A. (2000). Frequent ectopic recombination of virulence factor genes in telomeric chromosome clusters of P. falciparum. Nat. Cell Biol..

[101] Laporte D., Courtout F., Tollis S., Sagot I. (2016). Quiescent Saccharomyces cerevisiae forms telomere hyperclusters at the nuclear membrane vicinity through a multifaceted mechanism involving Esc1, the Sir complex, and chromatin condensation. Mol. Biol. Cell.

[102] Miné-Hattab J., Taddei A. (2019). Physical principles and functional consequences of nuclear compartmentalization in budding yeast. Curr. Opin. Cell Biol..

[103] Tanizawa H., Iwasaki O., Tanaka A., Capizzi J.R., Wickramasinghe P., Lee M., Fu Z., Noma K.-I. (2010). Mapping of long-range associations throughout the fission yeast genome reveals global genome organization linked to transcriptional regulation. Nucleic Acids Res..

[104] Hall I.M., Noma K.-I., Grewal S.I.S. (2003). RNA interference machinery regulates chromosome dynamics during mitosis and meiosis in fission yeast. Proc. Natl. Acad. Sci. USA.

